# Estimating the position and orientation of a mobile robot with respect to a trajectory using omnidirectional imaging and global appearance

**DOI:** 10.1371/journal.pone.0175938

**Published:** 2017-05-02

**Authors:** Luis Payá, Oscar Reinoso, Luis M. Jiménez, Miguel Juliá

**Affiliations:** 1 Department of Systems Engineering and Automation, Miguel Hernandez University, Elche, Alicante, Spain; 2 Q-Bot Limited, Block G, Riverside Business Centre, Bendon Valley, London, United Kingdom; Nanjing Normal University, CHINA

## Abstract

Along the past years, mobile robots have proliferated both in domestic and in industrial environments to solve some tasks such as cleaning, assistance, or material transportation. One of their advantages is the ability to operate in wide areas without the necessity of introducing changes into the existing infrastructure. Thanks to the sensors they may be equipped with and their processing systems, mobile robots constitute a versatile alternative to solve a wide range of applications. When designing the control system of a mobile robot so that it carries out a task autonomously in an unknown environment, it is expected to take decisions about its localization in the environment and about the trajectory that it has to follow in order to arrive to the target points. More concisely, the robot has to find a relatively good solution to two crucial problems: building a model of the environment, and estimating the position of the robot within this model. In this work, we propose a framework to solve these problems using only visual information. The mobile robot is equipped with a catadioptric vision sensor that provides omnidirectional images from the environment. First, the robot goes along the trajectories to include in the model and uses the visual information captured to build this model. After that, the robot is able to estimate its position and orientation with respect to the trajectory. Among the possible approaches to solve these problems, global appearance techniques are used in this work. They have emerged recently as a robust and efficient alternative compared to landmark extraction techniques. A global description method based on Radon Transform is used to design mapping and localization algorithms and a set of images captured by a mobile robot in a real environment, under realistic operation conditions, is used to test the performance of these algorithms.

## 1. Introduction

Map building and localization are two problems that have attracted significant attention in the field of mobile robots in recent years. The development of really autonomous mobile robots requires finding a relatively accurate and robust solution to these problems. Nowadays, omnidirectional vision systems have become a popular alternative to extract information from the environment, due to the big quantity of information they provide the robot with, since they have a 360 *deg* field of view in the horizontal plane. The images captured by these systems are named omnidirectional images. There are several configurations that permit obtaining such omnidirectional images [1]. Among them, catadioptric systems use external mirrors to widen the field of view of the camera. Some researchers have made use of the projection model of such systems to control the robot motion, as in [2] and [3], where a general catadioptric camera model is used to solve efficiently the problem of visual servoing of mobile robots.

In the present work, the only source of information used is a catadioptric vision system mounted on the robot. It is composed of a single-view camera pointing towards a convex hyperbolic mirror, with their axes aligned. This system provides omnidirectional images from the environment, as they contain information on a field of view of 360 *deg* around the mirror axis. Apart from this large field of view, catadioptric vision systems also permit carrying out other high level tasks, such as people detection and recognition, object identification, etc. However, using such highly dimensional data requires an effort to previously extract some relevant information from the scenes. This information must be useful to build a robust map and to estimate the position of the robot within this map.

Visual map building and localization can be addressed using two main frameworks, depending on how this most relevant information is extracted from the scenes and described. The first one consists in detecting some outstanding local features (i.e. landmarks) and describing them using an algorithm that provides some invariance to robot movements [4–7]. This approach has usually been used to create metric maps, which contains the position of some relevant features with respect to a coordinate system and permits estimating the position of the robot with geometrical accuracy, up to a specific error [8,9]. Despite its accuracy, these approaches present some drawbacks; the models tend to be complex and not easily understandable by a human operator and the necessary computational cost to extract, describe and track the local features along a set of images tends to be quite high. The second method consists in describing each scene as a whole, building a unique descriptor per image that contains information on its global appearance [10–12]. It often leads to models that can be handled more intuitively. However, such descriptors do not contain any metric information so they have been used traditionally to build topological maps, which contain information about some key locations of the environment and the connectivity relationships between them [13,14]. Despite their simplicity and compactness, this kind of models has proved to be adequate for mobile robot localization and navigation in controlled environments and the algorithms tend to present a reasonably low computational cost. Usually metric and topological models are combined in hybrid approaches, which try to arrive to a balance between the degree of detail and compactness of the map and the computational cost of the localization process [15]. A complete survey on robot mapping can be found in [16]. This survey shows how methods based on local features have reached a relative maturity; however, methods based on global appearance are worth a deeper study as there are many key aspects that have not been completely addressed. The present work focuses on the study of some of these aspects, as addressed in the following paragraphs.

On the one hand, many authors have considered sets of images captured on dense grid layouts to build complete models of the environment when using global appearance-methods [17,18]. However, the most common and straightforward way of capturing the set of images in a real application consists in tele-operating the robot or using any exploration algorithm [19] to define a free-of-obstacles trajectory that covers all the area to map. The robot would capture a set of omnidirectional images along this route and this visual information could be used to build a model or map of the environment. Solving the localization problem with such trajectory-like model and global appearance descriptors supposes a challenge, taking it into account the lack of metric information of such descriptors. The main contribution of this paper is related to this question. Some methods to describe the global appearance of omnidirectional scenes are presented and used to build a trajectory-like model of the environment and to estimate the position and orientation of the robot. The problem of visual control using such models is also addressed.

On the other hand, when a mobile robot has to move within a social environment, the scenes may be partially occluded by people and other robots that may be present in the environment [20]. The global appearance of the scenes may change substantially under these circumstances so the performance of the algorithms should be tested taking such phenomenon into account, as it will be present in most real applications.

In this work, some methods to describe the global appearance of omnidirectional images are presented and their performance to solve the robot localization problem is tested. These methods are based on the mathematical Radon Transform [21] and have been tested using our own trajectory-like set of omnidirectional images, captured in a real unstructured environment under real working conditions, and considering the eventual presence of severe noise and/or occlusions. The only source of information we will use to build a model of the environment and localize the robot are the omnidirectional images captured by a catadioptric vision system mounted on the robot.

The remainder of the paper is structured as follows. First, the methods we propose to describe the global appearance of scenes are outlined in section 2. After that, section 3 presents the approach to carry out robot localization and the experiments to validate this approach. Then, the problem of visual control is addressed in section 4, through an algorithm that permits deducing on which side of a route map the robot is, and the results of the experiments are shown. To finish, the experiments and contributions are discussed in section 5 and the conclusions and future works are addressed in section 6.

## 2. Global appearance descriptor of omnidirectional scenes

The key point of global appearance descriptors is the algorithm used to extract the most relevant information from each scene. This information must be useful to build a compact model of the environment that permits a subsequent estimation of the robot position. Some description algorithms can be found in the literature on this topic, based mainly on the Discrete Fourier Transform [22], on Principal Components Analysis [23], on the orientation of the edges in the scene [24,25] and on other relevant outstanding features, such as colour [26]. Previous works have shown how such descriptors may be used efficiently to build a model of the environment and to estimate the position and the orientation of the robot [14]. However, they tend to fail in real situations, when the robot has to cope with noise in the scenes and, mainly, when partial occlusions are present. Also, their performance in localization with respect to a trajectory-like model should be tested.

This section outlines some methods to describe the global appearance of omnidirectional images. Two families of methods are proposed to be studied: methods based on the Radon Transform and methods based on the Discrete Fourier Transform. With these methods, some feasible algorithms to create a model of the environment and to estimate the position of the robot will be proposed and evaluated along the next sections.

### 2.1. The Radon Transform

The Radon Transform (RT) consists in describing a function in terms of its line-integral projections. A line *c*_*l*_ can be parameterised with respect to its arc length *z* as *c*_*l*_ ≔ (*x*(*z*),*y*(*z*)) = ((*z* · *sinϕ* + *s* · *cosϕ*),(−*z* · *cosϕ* + *s* · *sinϕ*)) where *s* is the distance from *c*_*l*_ to the origin and *ϕ* is the angle between the normal vector to *c*_*l*_ and the *x*-axis. Taking this parametrisation into account, the RT of an image *f*(*x*,*y*) can be obtained with the next expression:

$$R\{f(x,y)\}=r_{im}(\phi ,s)=\int_{-\infty }^{+\infty }f(x(z),y(z))dz=\int_{-\infty }^{+\infty }f(z\cdot sin\phi +s\cdot cos\phi ,-z\cdot cos\phi +s\cdot sin\phi )dz$$ (1)

Where the coordinates of the new function are (*ϕ*,*s*) which can be considered as coordinates on the space of all lines in ℝ^**2**^. This way, a new 2D function is obtained through the integration of the original function *f*(*x*,*y*) along some sets of parallel lines with distance *s* among each of them and the origin and with different orientations *ϕ*. The size of the new descriptor is $r_{im}\in R^{M_{x}\times M_{y}}$, where *M*_*x*_ is the number of orientations considered $\phi =\{\phi_{1},\phi_{2},\ldots ,{\phi_{M}}_{x}\}$ and *M*_*y*_ is the number of parallel lines on each set.

The RT is invertible, and the inverse operation reconstructs a function from its line-integral projections. Taking its mathematical interpretation into account, the RT has been traditionally important in computer vision, in those situations where the original object *f*(*x*,*y*) is unknown and must be reconstructed from its line integral projections [27]. The most usual examples are medical imaging applications, such as computer axial tomography scan and magnetic resonance imaging. Apart from it, the RT has also been used in shape description and segmentation tasks [28].

**Fig 1:**
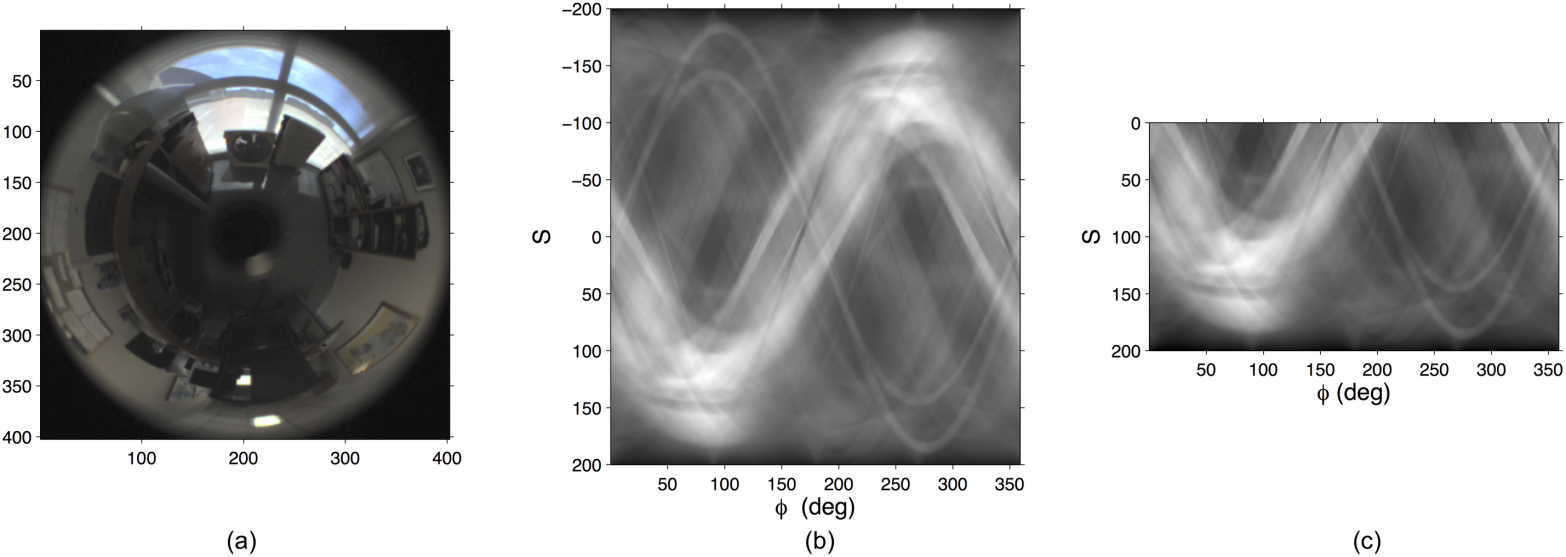
Radon Transform of an omnidirectional image. (a) sample omnidirectional image $f_{j}\in R^{N_{x}\times N_{x}}$, (b) Radon Transform of the sample image and (c) final RT descriptor of the sample image $r_{j}\in R^{360\times 0.5N_{x}}$.

The RT presents some interesting properties [29]. Symmetry and shift are especially important from the point of view of omnidirectional image description. Taking them into account, it is possible to obtain global appearance descriptors that permit estimating both the position and the orientation of the mobile robot. Fig 1(A) shows a sample omnidirectional image $f_{j}\in R^{N_{x}\times N_{x}}$, with *N*_*x*_ = 402 pixels and Fig 1(B) the RT of this image. To obtain this transform the distance between each pair of contiguous parallel lines in each set is equal to 1 pixel (i.e. *s* = 1,2,3,…,*N*_*x*_ *pixels* in Eq 1) and the orientation of these sets is chosen to cover the whole image (*ϕ* = 0,1,2,…,359 *deg* in Eq 1). The result is always an anti-symmetric matrix; thus the top half can be removed without loss of information. Fig 1(C) shows then the final descriptor of the original image $r_{j}\in R^{360\times 0.5N_{x}}$.

**Fig 2:**
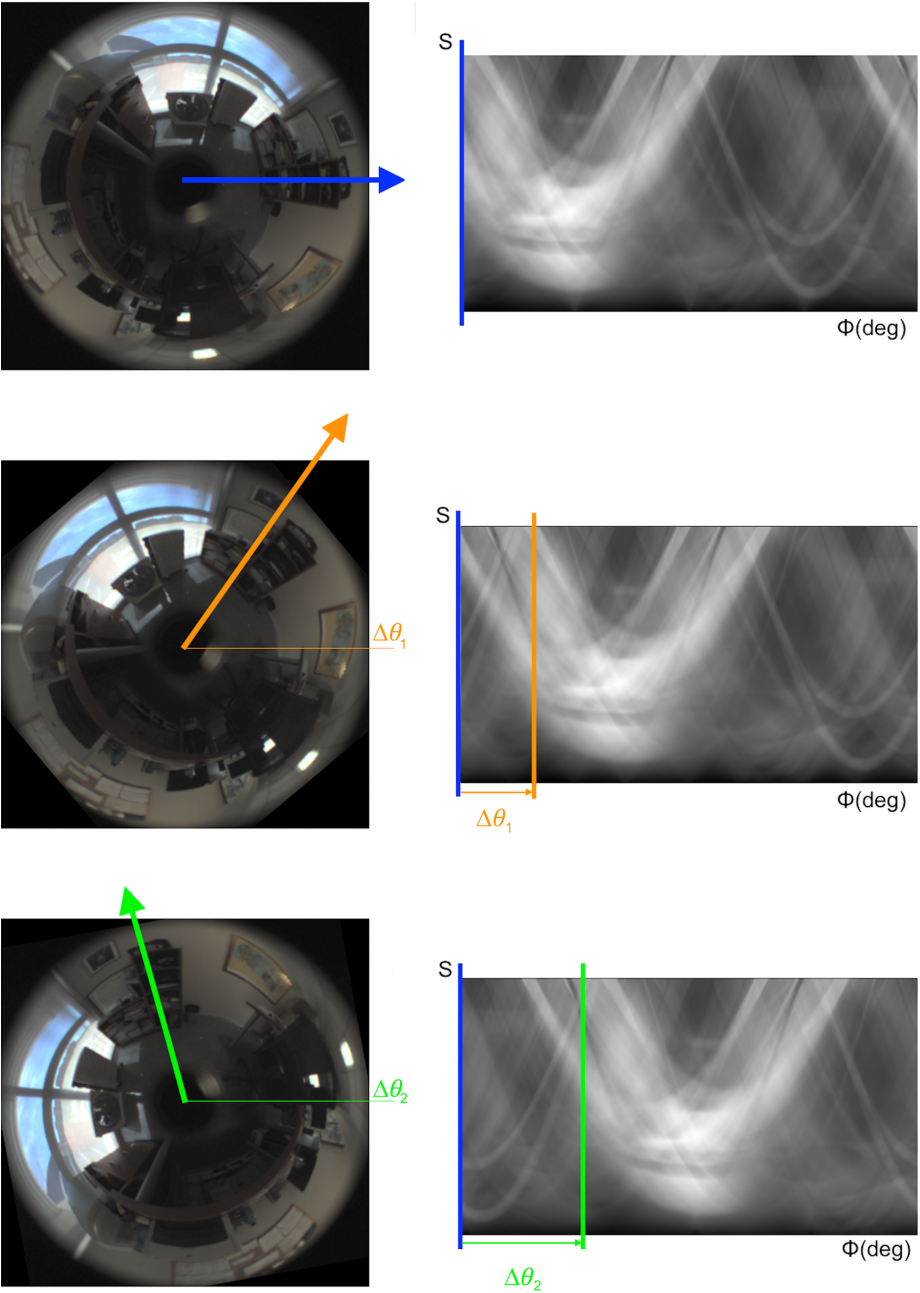
Shift property. The change of orientation of the robot on the ground plane has a shift effect on the columns of the RT descriptor.

On the other hand, the shift property is shown in Fig 2. This figure shows three omnidirectional images captured by the robot from the same floor position and with different orientations around the vertical axis. The figure shows clearly the effect of orientation on the Radon Transform. If the robot rotates Δ*θ deg* on the ground plane, the new descriptor presents the same information as the original descriptor but a shift of columns equal to *d* = Δ*θ* · *M*_*x*_/360, with Δ*θ* measured in *deg*.

Taking these facts into account, the RT descriptor contains information both on the global appearance of the environment and on the robot orientation. Starting from an omnidirectional image $f_{j}\in R^{N_{x}\times N_{x}}$ a global descriptor $r_{j}\in R^{360\times 0.5N_{x}}$ can be built by calculating its Radon Transform and retaining the bottom half. The subsequent sections study the use of this descriptor to estimate both the position and the orientation of the robot.

### 2.2. The Fourier Signature

The Discrete Fourier Transform (DFT) is a classical method that has been used by many researchers to extract the most relevant information from scenes. It permits expressing the information in the frequency domain and presents several interesting properties in image description [14]. Some authors have made use of different algorithms based on the DFT to solve a wide range of tasks. For example, the Fractional Fourier Transform has proved to be effective in the implementation of computer-aided diagnosis systems, using brain magnetic resonance images [30] or mammographic images [31]. Also, the Fourier Signature, has shown efficiency to carry out mapping and localization tasks using panoramic images [22].

In this work, we make use of the Fourier Signature (FS), described first in [22]. It is defined as the matrix composed of the one-dimensional DFT of each row in the original image. It contains useful information on the global appearance of the scene and, when applied to panoramic scenes, it presents rotational invariance (i.e. it contains the same information independently on the robot orientation on the ground plane).

When the FS of a panoramic image $f_{j}(x,y)\in R^{N_{x}\times N_{y}}$ is calculated, a new matrix $F_{j}(u,y)\in C^{N_{x}\times N_{y}}$ is obtained (*u* is the frequency variable, measured in *cycles/pixel*), where the main information of the original image is concentrated in the low frequency components of each row (so only the *k* first columns can be retained, having a compression effect). This new matrix $d_{j}(u,y)\in C^{N_{x}\times k}$ can be decomposed into a magnitudes matrix *A*_*j*_(*u*,*y*) = |*d*_*j*_(*u*,*y*)| and an arguments matrix Φ_*j*_(*u*,*y*). Based on the shift property of the DFT, when two panoramic images have been captured from the same position but having the robot different orientation, both images have the same magnitudes matrix and the arguments matrices permit obtaining the relative robot orientation [14]. This property allows us to use first the magnitudes matrix to estimate the position of the robot (as it presents rotational invariance) and then, the arguments matrix to estimate the relative orientation of the robot.

## 3. Creating a visual model of the environment and estimating the position of the robot

This section focuses on the problem of map creation and localization. The mobile robot is equipped with a catadioptric vision system on its top, which provides it with omnidirectional images from the environment. This is the only source of information that will be used both to build a model of the environment and to estimate the position of the robot (nor odometry neither laser or other sensory data will be used). This way, the final model will be a topological model since it will contain information of some localizations or nodes (represented as omnidirectional scenes) along with their connectivity relations (but no metric data). In the next subsections, first, the methods developed to create a visual model and to estimate the position of the robot are presented and then, the experiments carried out to validate the approach are detailed.

### 3.1. Topological mapping and absolute localization methods

In this work we propose solving the mapping and localization problem in two consecutive steps: first a topological model is built and then, this model is used to estimate the current (unknown) position and orientation of the robot in an absolute way (i.e. no information on the previous position of the robot is used to estimate its current position).

First, to build a model, some visual information from the environment is necessary. During this learning phase, the robot has to traverse a trajectory that covers the entire environment to map (either in a teleoperated way or autonomously, according to any exploration algorithm). Along it, the robot captures a set of omnidirectional images *I* = {*f*_1_,*f*_2_,…,*f*_*n*_} where $f_{j}\in R^{N_{x}\times N_{x}}$. To build the model, this set of images is transformed into a set of descriptors, one per original scene. As a result, the nodes of the map will be a set of descriptors *D* = {*d*_1_,*d*_2_,…,*d*_*n*_} where, in general, $d_{j}\in C^{M_{x}\times M_{y}}$. No metric information is included in this model. Each descriptor *d*_*j*_ must contain enough information to permit estimating the position and orientation of the robot with robustness and computational efficiency. Otherwise, the model would not be useful.

Second, once the map is built, the robot must be able to estimate its current (unknown) position with respect to this model. The absolute localization problem assumes the robot has no information on its previous position. This way, it has to compare the visual information it currently captures with all the information previously stored in the model. The localization is addressed in this paper as an *image retrieval* problem [32]; the robot captures an image from an unknown position and the objective consists in detecting which of the images previously stored in the map is the most similar one.

With this aim, the robot captures a new test image at time instant *t*, *f*_*t*_, describes it to obtain *d*_*t*_ and compares it with all the nodes of the map. As a result, the position and the orientation of the robot must be estimated. With this aim, the distance between *d*_*t*_ and the nodes of the map *D* = {*d*_1_,*d*_2_,…,*d*_*n*_} is calculated, obtaining the distances vector $l_{t}=\{{l_{t}}_{1},{l_{t}}_{2},\ldots ,{l_{t}}_{n}\}$ where ${l_{t}}_{j}=dist\{d_{t},d_{j}\}$ according to any distance measure. The node that presents the minimum distance $d_{t}^{nn}/t=arg\;\min_{j}l_{tj}$ (nearest neighbour) is considered the corresponding position of the robot. Afterwards, comparing $d_{t}^{nn}$ with *d*_*t*_, the robot orientation must be estimated.

Taking the properties of the Radon Transform into account, three methods are proposed to create the nodes *D* = {*d*_1_,*d*_2_,…,*d*_*n*_} and to solve the localization problem. In all cases, first we detail the kind of information each node descriptor *d*_*j*_ contains and second we give details about the localization process.

#### a) Method 1

The RT descriptor is not invariant against rotation *per se*, because the information contained in the descriptor changes when the robot rotates. As shown in Fig 2, a pure rotation of the robot produces a circular shift of the columns of the RT descriptor. This effect must be taken into account during the implementation of a localization method. In method 1 the FS is used to transform the RT descriptor, in order to obtain a new descriptor which is completely invariant against rotation. More concisely, for each omnidirectional image *f*_*j*_, the RT descriptor is obtained $r_{j}\in R^{M_{x}\times M_{y}}$ and then the FS of this descriptor is calculated. As a result, each node *d*_*j*_,*j* = 1,…,*n* contains two matrices: the magnitudes matrix $A_{j}\in R^{M_{x}\times k}$ and the arguments matrix $\Theta_{j}\in R^{M_{x}\times k}$. The first one contains information on the appearance of the environment and is independent on the robot orientation thus it permits estimating the position of the robot. The second matrix permits estimating the robot orientation. This is an advantage, as both problems are decoupled, and the estimation of the position and the orientation can be done sequentially.

To carry out the localization process, when the robot captures a new test image *f*_*t*_ from an unknown position, first, the RT descriptor is obtained $r_{t}\in R^{M_{x}\times M_{y}}$. Second, the FS of this descriptor is calculated, and the magnitudes matrix $A_{t}\in R^{M_{x}\times k}$ and the arguments matrix $\Theta_{t}\in R^{M_{x}\times k}$ are obtained. Once these matrices are available, ***A***_*t*_ is compared with the matrices ***A***_*j*_,*j* = 1,…,*n* to solve the *image retrieval* problem. With this aim, the distance between ***A***_*t*_ and each matrix ***A***_*j*_ is obtained, and the node that presents the minimum distance (nearest neighbour) is considered the corresponding position of the robot.

After that, the arguments matrix $\Theta_{t}^{nn}$ of the nearest neighbour node $d_{t}^{nn}$ is compared with ***Θ***_*t*_ to estimate the relative orientation of the robot. The shift theorem of the DFT (Eq 2) is used to do it, following the next steps. We consider the robot captures an omnidirectional image from a specific position and calculates its RT, obtaining $r_{1}\in R^{M_{x}\times M_{y}}$. We represent each row of this matrix as a sequence {*a*_*n*_} and the *k*-th component of the DFT of this row is $F{\left(\left\{a_{n}\right\}\right)}_{k}=A_{k}$. If we consider now a second image, captured from the same position but having the robot a different relative orientation *Δθ* (*deg*), the RT of this image, $r_{2}\in R^{M_{x}\times M_{y}}$ will contain the same information than ***r***_1_ but with a circular shift of columns equal to *q* = *Δθ*° · *M*_*y*_/360. This way, each row of ***r***_2_ can be represented as the shifted sequence {*a*_*n*−*q*_}. According to the shift theorem, the DFT of this shifted sequence can be calculated as:

$$F{\left(\left\{a_{n-q}\right\}\right)}_{k}=A_{k}\cdot e^{-j\cdot \frac{2\pi qk}{N_{y}}};\;k=0,1,\ldots ,M_{y}-1$$ (2)

where F is the DFT operand, $j=\sqrt{-1}$ and *A*_*k*_ are the components of the DFT of the original row (without shift). We take advantage of this theorem to estimate the relative orientation of the robot. Starting from the arguments matrix of the test image ***Θ***_*t*_, a set of artificial rotations is generated, using Eq 2, to cover the whole circumference. The resulting arguments matrix after each artificial rotation is compared with the nearest neighbour arguments matrix $\Theta_{t}^{nn}$. To do this, the Hadamard product between matrices is calculated. The orientation of the rotated matrix that produces the maximum Hadamard product is the estimated orientation of the robot.

#### b) Method 2

This method is an adaptation of method 1 to try to optimize the computational cost of the comparisons between descriptors. The method starts following the same steps: the RT descriptor of each omnidirectional image *f*_*j*_ is calculated, obtaining $r_{j}\in R^{M_{x}\times M_{y}}$ and then the FS of them is calculated, obtaining the magnitudes matrices $A_{j}\in R^{M_{x}\times k}$ and the arguments matrices $\Theta_{j}\in R^{M_{x}\times k}$. Once the magnitudes matrix of each image has been obtained, the average value of each column is calculated, obtaining the localization descriptor ${\vec{a}}_{j}\in R^{1\times k},j=1,\ldots ,n$. The orientation descriptor is the same as in method 1. As a result, each node *d*_*j*_ contains two components: the vector ${\vec{a}}_{j}$ and the arguments matrix ***Θ***_*j*_.

The image retrieval problem is solved now with the descriptors ${\vec{a}}_{t}$ and ${\vec{a}}_{j},\;j=1,\ldots ,n$. The estimation of the orientation is carried out as in method 1. Since the size of the position descriptor has been substantially reduced, the estimation of the position is expected to be a quicker process with this method.

#### c) Method 3

This method consists in describing each image directly through its RT descriptor $r_{j}\in R^{M_{x}\times M_{y}}$ and using the Phase Only Correlation (POC) to compare two descriptors. This way, in this case, each node *d*_*j*_,*j* = 1,…,*n* contains only one component, ***r***_*j*_.

The image retrieval problem and orientation estimation are solved through the POC operation [33]. This operation is carried out in the frequency domain and provides us with a correlation coefficient that permits estimating the similitude between two scenes that may present a relative offset (and this relative offset can also be estimated thanks to POC [34]). POC is a correlation method based upon the inverse Fourier transform of the phase difference between two images. Therefore, it can measure both the similitude between two images and the shift between them. The use of the phase information has proved to be a robust choice to provide immunity to various types of noise or other image distortions [33]. The POC is based on the fact that the information of the shift between two images resides in the phase of the cross power spectrum.

In general, the POC between two matrices *m*_1_(*x*,*y*) and *m*_2_(*x*,*y*) is defined according to Eq 3:

$$C_{m_{1}m_{2}}(x,y)=F^{-1}\left\{\frac{M_{1}(u,v)\cdot M_{2}^{*}(u,v)}{\left|M_{1}(u,v)\cdot M_{2}^{*}(u,v)\right|}\right\}$$ (3)

where ***M***_1_(*u*,*v*) is the two dimensional DFT of *m*_1_, $M_{2}^{*}(u,v)$ is the conjugated two dimensional DFT of *m*_2_ and $F^{-1}$ is the 2D inverse DFT operand.

The result of this operation is a matrix $C_{m_{1}m_{2}}(x,y)$ which has the same dimensions than *m*_1_ and *m*_2_. It contains information on the similitude between these matrices and also on the relative shift between them. First, the component of this matrix that takes the maximum value, $max\{C_{m_{1}m_{2}}(x,y)\}$, is a coefficient that takes value in the interval [0,1] and measures the similitude between the two matrices *m*_1_ and *m*_2_. It is invariant against shifts of *m*_2_ with respect to *m*_1_ (both in files and/or in columns). Taking this fact into account, in this work, we consider as distance measure:

$$dist\{m_{1},m_{2}\}=1-max\{C_{m_{1}m_{2}}(x,y)\}$$ (4)

Second, the shift of the second matrix *m*_2_ with respect to *m*_1_ can be obtained with the next expression:

$${\left(\Delta x,\Delta y\right)}_{m_{2}m_{1}}=arg\;\max_{\left(x,y\right)}\left\{C_{m_{1}m_{2}}(x,y)\right\}$$ (5)

In this work, the two matrices to compare are the Radon Transforms of two images $r_{f1}(\phi ,s)=R\{f_{1}(x,y)\}$ and $r_{f2}(\phi ,s)=R\{f_{2}(x,y)\}$. Taking the shift property into account (Fig 2), the relative orientation of the robot between the positions 1 and 2 is:

$$\theta_{21}=\theta_{2}-\theta_{1}={\Delta \phi }_{r_{2}r_{1}}=arg\;\max_{\phi }\left\{C_{r_{1}r_{2}}(\phi ,s)\right\}$$ (6)

where:

$$C_{r_{1}r_{2}}(\phi ,s)=F^{-1}\left\{\frac{R_{f1}(u,v)\cdot R_{f2}^{*}(u,v)}{\left|R_{f1}(u,v)\cdot R_{f2}^{*}(u,v)\right|}\right\}$$ (7)

$$R_{f1}(u,v)=F\{r_{f1}(\phi ,s)\};\;R_{f2}(u,v)=F\{r_{f2}(\phi ,s)\}$$ (8)

**Fig 3:**
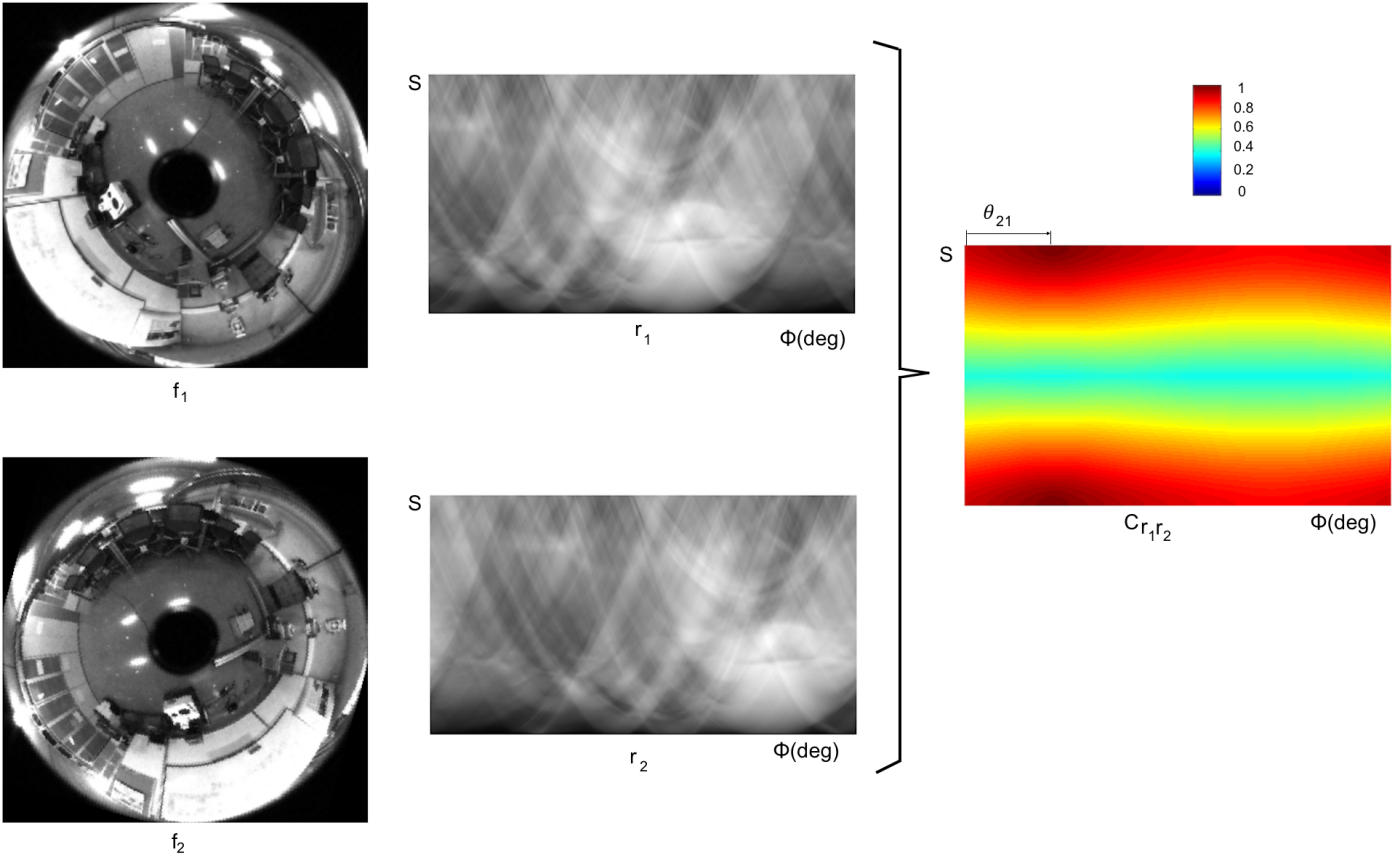
Phase only correlation. Result of the POC operation between two RT descriptors ***r***_1_ and ***r***_2_. The original images *f*_1_ and *f*_2_ were captured on the same point of the floor plane with orientations *θ*_1_ and *θ*_2_ respectively. The RT descriptors of these images are ***r***_1_ and ***r***_2_ respectively. The result of the POC operation between ***r***_1_ and ***r***_2_ is the matrix $C_{r_{1}r_{2}}$, whose elements take values between 0 and 1. These values are shown in a colour scale. The maximum value in $C_{r_{1}r_{2}}$ is a measure of similitude between ***r***_1_ and ***r***_2_ (the higher value the maximum component takes, the most similar both matrices are) and it is independent on the change of orientation. The position of the maximum value permits estimating the relative orientation of the robot when capturing both initial images *θ*_21_ = *θ*_2_ − *θ*_1_.

Fig 3 shows two sample omnidirectional images, captured from the same point on the floor but with a change of orientation *θ*_21_ between them. Their RT descriptor and the POC operation between descriptors is shown. The maximum value of the POC is in the first row and in the column *θ*_21_.

### 3.2. Experiments

In this subsection, a study is carried out to evaluate the performance of the proposed methods in an absolute localization task. First, the sets of images used to develop the experiments are described. Then, the evaluation is carried out to find out the performance of each method and the results of the experiments are shown.

**Fig 4:**
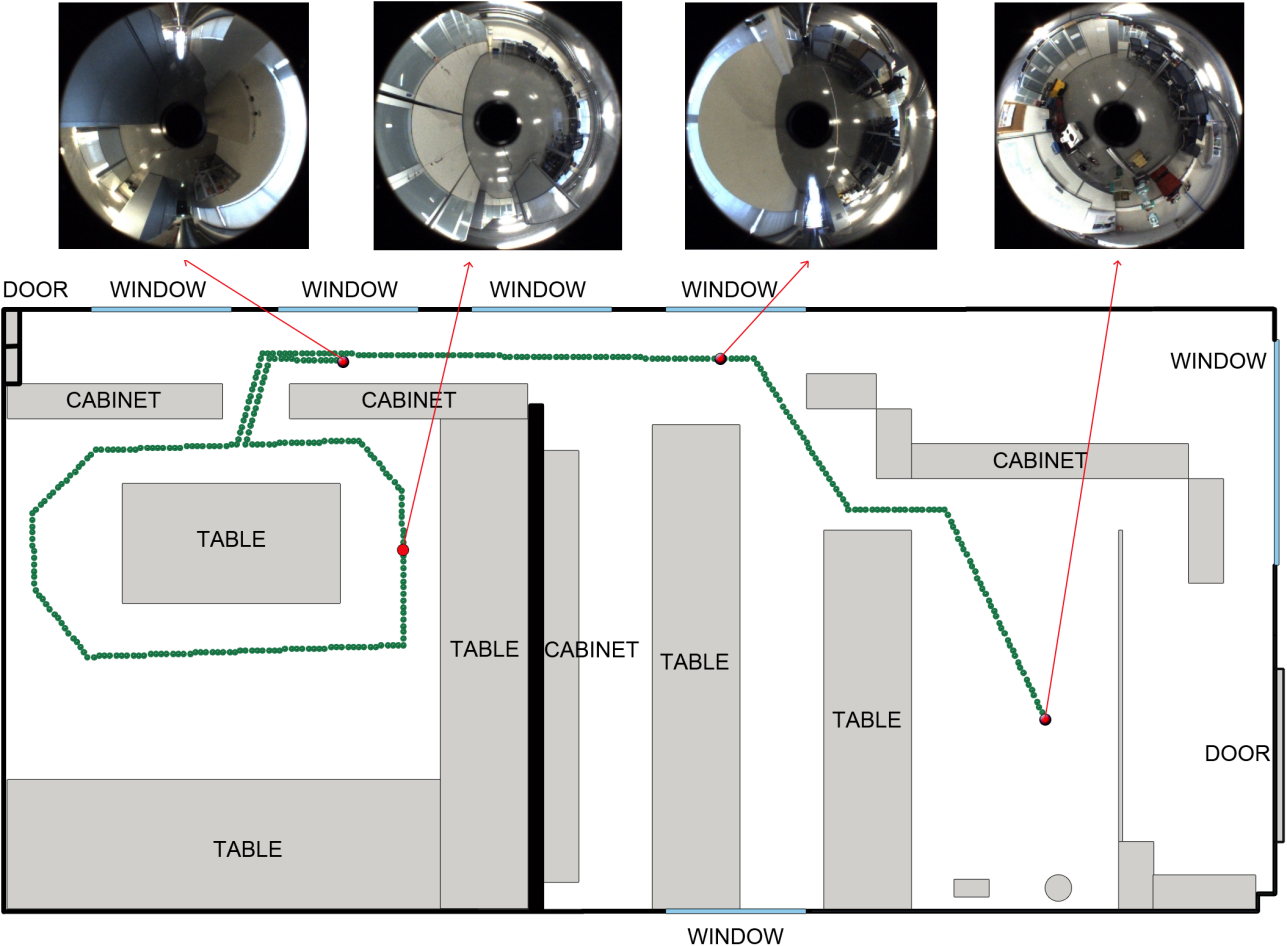
Set of images. Trajectory followed by the robot to capture the whole set of images. The capture points are shown as green dots. Some sample omnidirectional images are shown.

The experiments have been performed with a database captured by ourselves, using an Imaging Source DFK 21BF04 camera, which takes pictures of a hyperbolic mirror (Eizoh Wide 70). The mirror is mounted over the camera, with its axis aligned with the camera optic axis. The whole database contains 400 images that were captured while the robot went through a previously defined trajectory in a laboratory area, at Miguel Hernández University (Spain). The distance between each pair of consecutive images is equal to 20cm. The route covers a distance equal to 80m and the environment where the images were captured is very prone to visual aliasing (the visual appearance of some images that have been captured in different rooms may be similar). Fig 4 shows a bird eye’s view of the positions where the images were captured and some sample omnidirectional images. This database is available from [35]. To carry out the localization experiment, the images have been divided into two groups: the *training set*, which is composed of 200 images, taking one every two and the *test set*, composed of the rest of images.

To carry out the experiment, first the descriptor of each training scene has been obtained and stored to compose the map. Then, the absolute localization problem has been solved with all the test images. Four different distance measures have been tested along with methods 1 and 2: *d*1 is the *cityblock* distance, *d*2 is the Euclidean distance, *d*3 is the correlation distance and *d*4 is the cosine distance. With method 3, the distance measure used is POC. Table 1 shows the definition of each type of distance. In these definitions, $\vec{a}\in R^{l\times 1}$ and $\vec{b}\in R^{l\times 1}$ are the two data vectors to compare, whose components are respectively *a*_*i*_,*b*_*i*_,*i* = 1,…,*l*.

**Table 1:** Distance measures.

Distance measure	Type of distance	Mathematical expression
*d*1	*Cityblock*	$d_{1}(\vec{a},\vec{b})=\sum_{i=1}^{l}\left|a_{i}-b_{i}\right|$
*d*2	Euclidean	$d_{2}(\vec{a},\vec{b})=\sqrt{\sum_{i=1}^{l}{\left(a_{i}-b_{i}\right)}^{2}}$
*d*3	Correlation	$d_{3}(\vec{a},\vec{b})=1-\frac{{\vec{a}}_{d}^{T}\cdot {\vec{b}}_{d}}{\left|{\vec{a}}_{d}|\cdot |{\vec{b}}_{d}\right|}$ where: ${\vec{a}}_{d}=[a_{1}-\bar{a},\ldots ,a_{l}-\bar{a}];\;\bar{a}=\frac{1}{l}\sum_{j}a_{j}$ ${\vec{b}}_{d}=[b_{1}-\bar{b},\ldots ,b_{l}-\bar{b}];\;\bar{b}=\frac{1}{l}\sum_{j}b_{j}$
*d*4	Cosine	$d_{4}(\vec{a},\vec{b})=1-\frac{{\vec{a}}^{T}\cdot \vec{b}}{\left|\vec{a}|\cdot |\vec{b}\right|}$

Distance measures used along with methods 1 and 2 to compare descriptors in the localization process.

During the experiments, the eventual presence of noise and/or occlusions in the test images has been considered, since these are two common phenomena that may be present in a real localization application.

**Fig 5:**
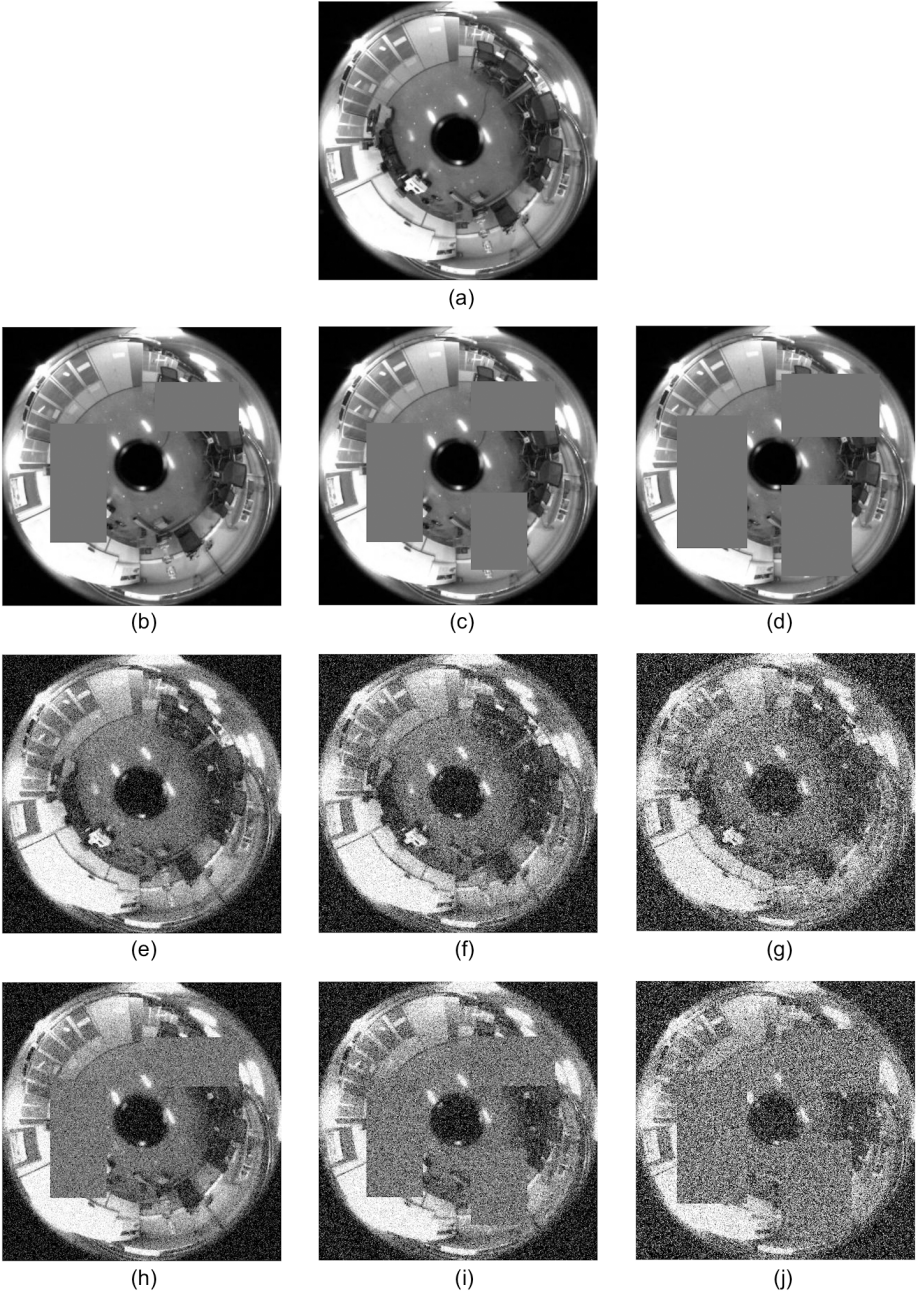
Occlusions and noise on the test images. (a) Original sample test image with (b), (c), (d) different increasing levels of partial occlusion; (e), (f), (g) increasing levels of Gaussian noise and (h), (i), (j) increasing levels of simultaneous occlusion and noise.

First, three occlusion levels have been considered: 1) 20%, 2) 25% and 3) 35% of the test image occluded. Second, Gaussian noise with three different variances has been superposed to the test images; 1) *σ*^2^ = 0.025, 2) *σ*^2^ = 0.05 and 3) *σ*^2^ = 0.1. At last, both effects have been combined on the test images, considering the next three cases with respect to the percentage of occlusion and variance of the Gaussian noise: 1) 20% and *σ*^2^ = 0.025, 2) 25% and *σ*^2^ = 0.05 and 3) 35% and *σ*^2^ = 0.1. Fig 5(A) shows a sample test image and the effect of the three levels of occlusion considered (Fig 5(B), 5(C) and 5(D)), the effects of the Gaussian noise with different variances (Fig 5(E), 5(F) and 5(G)) and the combined effect of both occlusions and noise (Fig 5(H), 5(I) and 5(J)).

**Fig 6:**
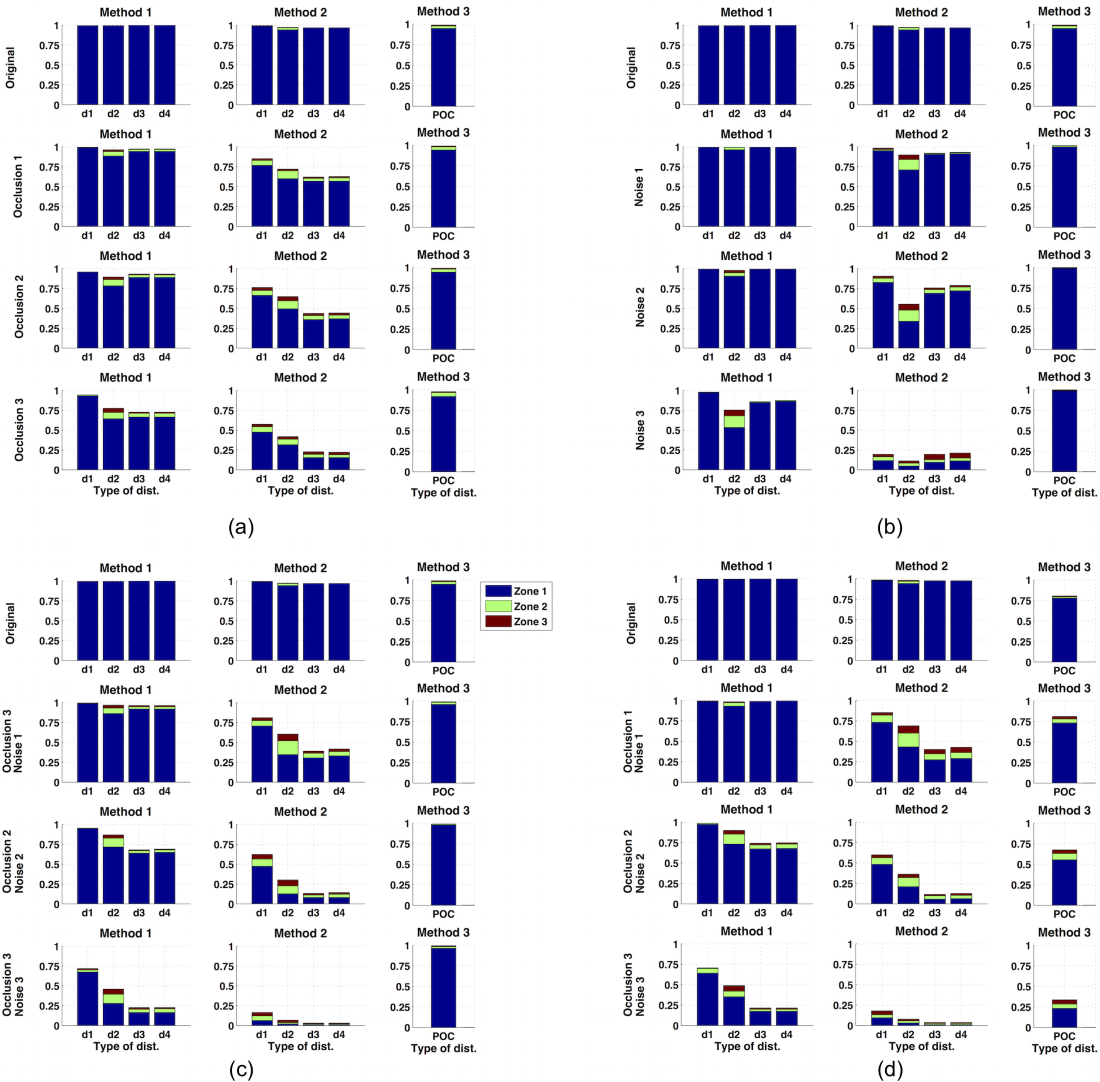
Localization results. Percentage of correct localizations when (a) occlusions are present in the test images (b) noise is considered, (c) simultaneous occlusions and noise are considered and (d) the robot presents any orientation in the test position and also simultaneous occlusions and noise.

Taking all these facts into account, the localization results obtained after the experiments are shown in Fig 6. The bars that appear in this figure show the percentage of correct decisions of each algorithm (expressed on a per-unit basis). Three precision measures have been considered. The first one considers a correct decision when the algorithm returns one of the two true nearest positions (zone 1) and is shown with a blue bar; the second one when the algorithm returns one of the four true nearest positions (zone 2) and is shown with a green bar and the last one, one of the six true nearest positions (zone 3) and is shown with a red bar.

First, Fig 6(A) shows the localization results when partial occlusions are present on the test image. In this experiment, the orientation of each test image is the same than the orientation of the nearest map image. The method 2 tends to degrade quickly as the level of occlusions increases. In this case the method 1 with the *cityblock* distance and the method 3 present the most stable behaviour and they have a percentage of correct answers around 98% with the higher level of occlusions.

Second, Fig 6(B) shows the localization results when noise appears on the test images. The orientation of the test images is again equal to the orientation of the nearest map image. As shown in the figure, the method 1 with the *cityblock* distance and the method 3 present excellent results as the percentage of correct localizations is 100% even in the case of the highest level of noise.

Third, the simultaneous presence of noise and occlusions is considered. The localization results obtained in this case are presented in Fig 6(C). Method 3 presents a percentage of correct localizations near 100% and method 1 with the *cityblock* distance around 75%. These two configurations clearly outperform the other methods. The results provided by method 2 are significantly low in the case of the maximum level of simultaneous occlusion and noise.

To complete the experiment, the effect of changes in the robot orientation has been tested. With this aim, the localization experiment is repeated but considering the robot has different orientations on each test position. The range of orientations considered for each test image is *θ*_*t*_ = [0,10,20,30,…,350] *deg*. After each experiment, the relative orientation of the robot is calculated, comparing each test image with its nearest neighbour. Fig 6(D) shows the localization results in this case, considering also the simultaneous presence of occlusions and noise. This figure shows how method 3 tends to perform worse when the orientation of the robot changes. However, the comparison with Fig 6(C) shows how method 1 presents the same behaviour independently on the robot orientation so this is the method that best represents the robot position with rotational invariance. In general, the best results have been obtained with method 1 using the *cityblock* distance, with around 75% of correct choices when considering changes in orientation and the higher level of simultaneous noise and occlusions and 100% of correct choices when considering changes in orientation and the second level of simultaneous noise and occlusions. The results of this experiment show that, despite the challenging problem (because the visual appearance of the scenes has been seriously compromised), is it possible to build a descriptor based on the Radon Transform that describes visually the robot position robustly and with rotational invariance.

**Fig 7:**
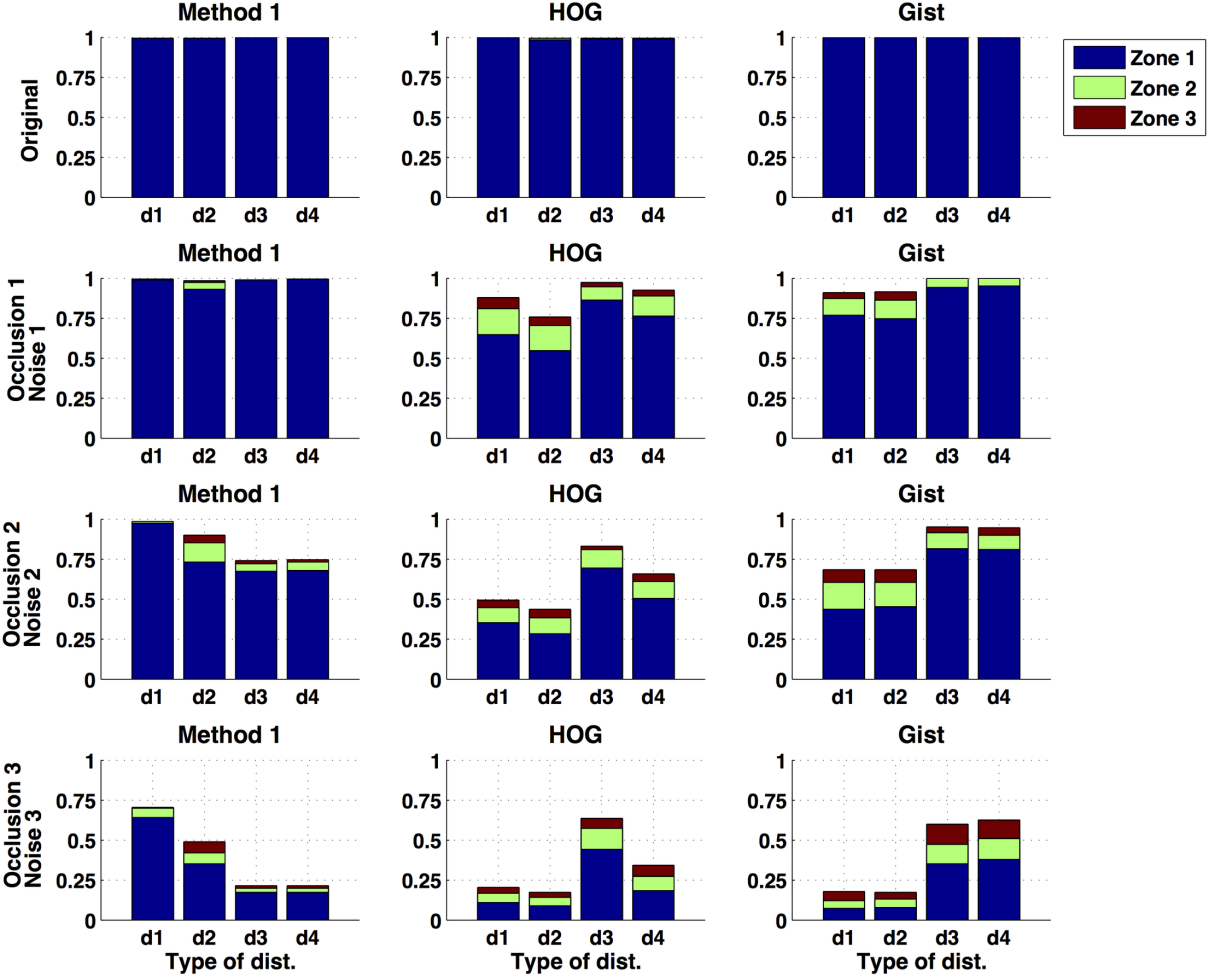
Localization results. **Comparative between the method 1, HOG and *gist***. Percentage of correct localizations when the robot presents any orientation in the test position and also simultaneous occlusions and noise.

Once the performance of the proposed methods in position estimation has been tested, we compare it with the performance of two well-known global appearance descriptors: Histograms of Oriented Gradients (HOG) and *gist*. They have been used previously in map building tasks, and a complete description can be found in [18]. Fig 7 shows the comparative results obtained with our method 1, HOG and *gist* in position estimation, when the robot presents any orientation in the test position, and with the simultaneous presence of noise and occlusions (it is the equivalent to Fig 6(D)). Only the results of method 1 have been included in this figure since it is the method that has provided the best localization results. Fig 7 shows that both HOG and *gist* provide excellent results when the test image does not present neither occlusions nor noise. However, their performance degrades quicker as the level of occlusions and noise increase, compared to the performance of the method 1. In these cases, our method 1 along with the *cityblock* distance proves to be more robust and outperforms both HOG and *gist*. It presents the best localization results in any zone, specially in zone 1 (the most restrictive one).

Apart from the accuracy in position estimation, it is also interesting to study the necessary processing time to solve the localization problem. Table 2 shows the average time to estimate the position of the robot, depending on the method and type of distance used. To measure this time, the experiments have been carried out using Matlab running on a 2 x 2.4 GHz 6-Core Intel Xeon processor. Initially, the model of the environment is available (i.e. the *n* descriptors of the images that compose the model, D = {d_1_,d_2_,…,d_n_} are calculated previously, where *n = 200*). This way, the position estimation time is the necessary time to make all the calculations since the omnidirectional test image is available: to describe it, to compare the descriptor with the *n* descriptors stored in the model and to detect the nearest neighbour. In general, the method 2 is the quickest one, as expected. Nevertheless, the method 1 along with distances 1 and 2 (*cityblock* and Euclidean) also presents a good processing time, lower than in the case of HOG and *gist*. This way, the method 1 with distance 1 presents a higher accuracy and an improved computation time compared to the two reference methods studied.

**Table 2:** Computation time of the position estimation process.

Method	Distance	t (msec)
RT–Method 1	*d1*	137
	*d2*	136
	*d3*	245
	*d4*	235
RT–Method 2	*d1*	103
	*d2*	102
	*d3*	103
	*d4*	103
RT–Method 3	*POC*	394
HOG	*d1*	152
	*d2*	151
	*d3*	152
	*d4*	152
*gist*	*d1*	197
	*d2*	196
	*d3*	197
	*d4*	197

**Fig 8:**
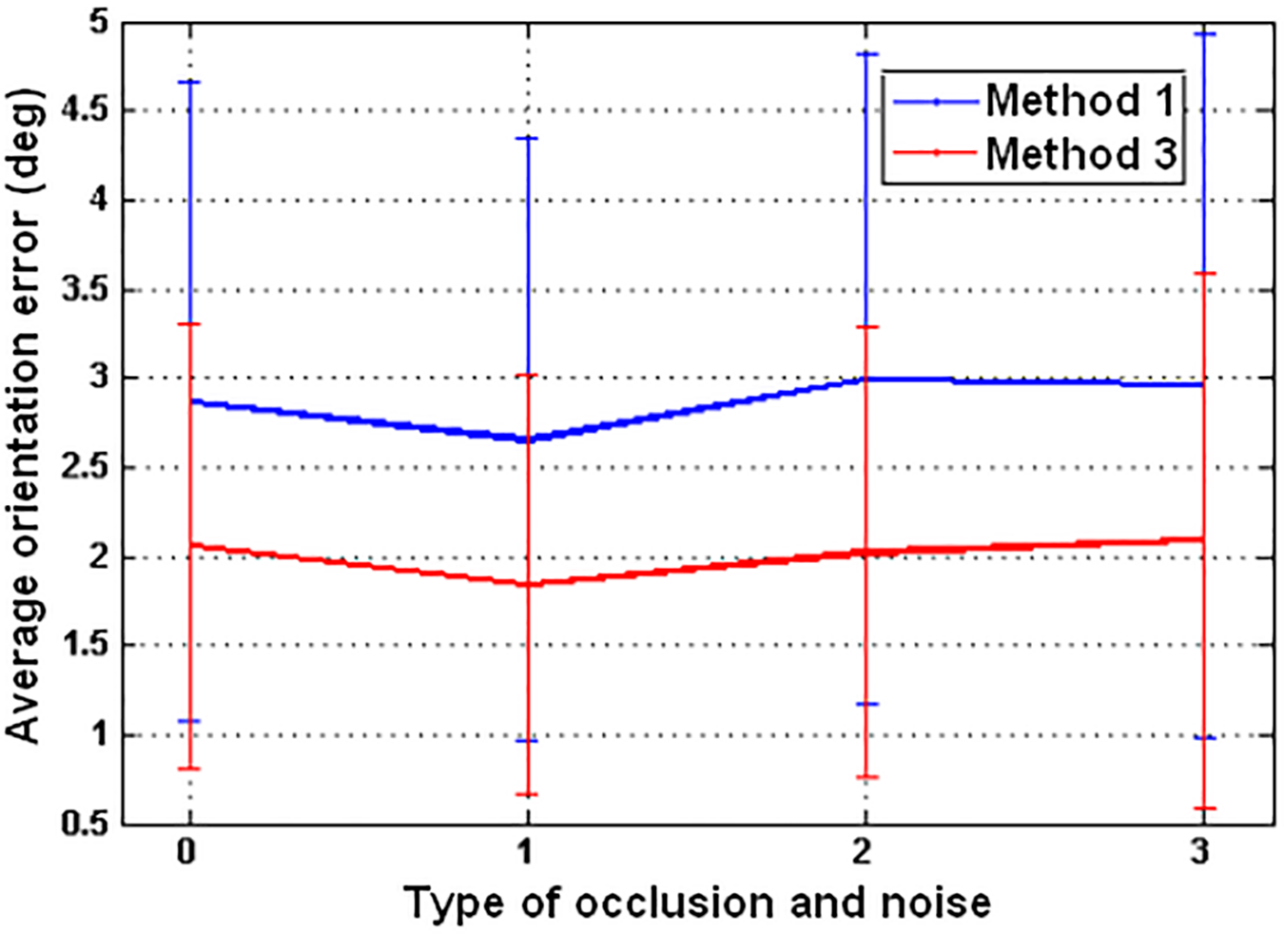
Orientation estimation results. Average orientation error obtained with method 1 and *cityblock* distance and with method 3. Simultaneous occlusions and noise are considered in the test images.

Apart from the position, it is also interesting to estimate the robot orientation. With this aim, the set of test images that include different robot orientations can be used. Fig 8 shows the orientation estimation results. Method 1 with the *cityblock* distance and method 3 are compared in this figure, as these are the two methods that have presented the best localization results (including the presence of noise and occlusions). To obtain this figure, the descriptor of each test image has been compared with the descriptor of the geometrically closer map image and, as a result, the relative orientation between the test and the map image has been calculated. In the vertical axis, the average orientation error is shown, and in the horizontal axis, several levels of simultaneous noise and occlusion are considered. ‘0’ represents no noise nor occlusion, ‘1’ simultaneous 20% occlusion and *σ*^2^ = 0.025 Gaussian noise, ‘2’ 40%, *σ*^2^ = 0.05 and ‘3’ 40% and *σ*^2^ = 0.1.

Fig 8 shows that both methods present a quite stable behaviour in the orientation estimation, independently on the added occlusions or noise. The method 1 with the *cityblock* distance presents an average error around *3 deg* and method 3 around *2 deg*, even in the case of the higher level of simultaneous occlusion and noise. Taking this fact into account, the Radon Transform is able to generate a descriptor that not only describes visually the robot position robustly but also is able to estimate the robot orientation with accuracy. The average processing time to estimate the orientation with method 1 is equal to 115 msec. The processing time with method 3 is included in Table 2, because when POC is used, this operation is able to estimate both the position of the robot and the relative orientation with the same calculations. This way, method 3 takes 394 msec to estimate both the position and the orientation of the robot, and method 1 with distance *d1* takes 137 msec to estimate the position plus 115 msec to estimate the orientation.

**Fig 9:**
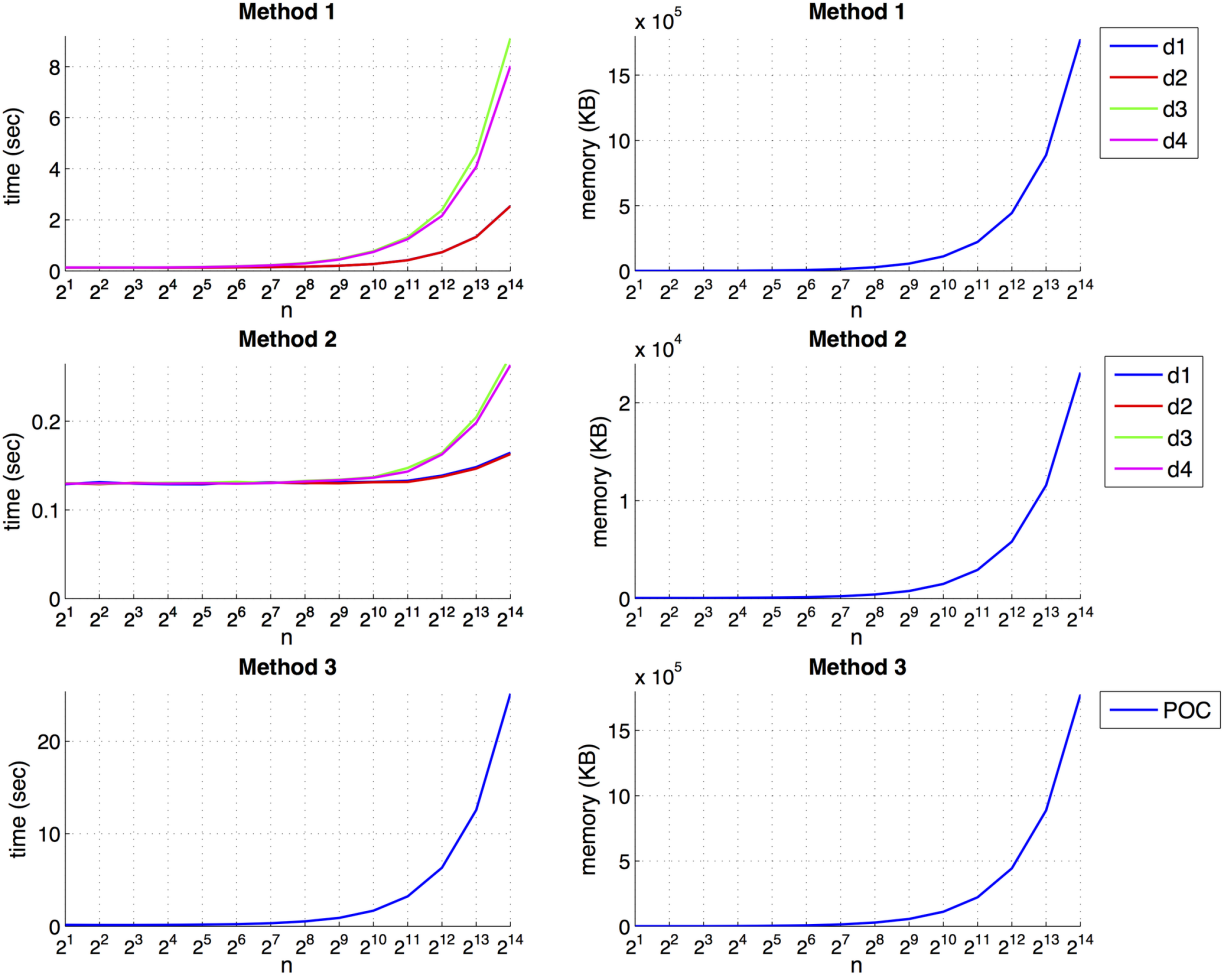
Processing time and memory requirements of the proposed methods. Average localization time and memory requirements of each method versus the size of the model (*n* is the number of images that the model contains).

To finish this section, the scalability of the position estimation algorithms (methods 1, 2 and 3) is tested. All the methods have been implemented as a single process running on a single processor. To study their scalability, we consider different sizes of the model used to estimate the position of the robot and we study the performance of each method with respect to the processing time and memory requirements. The results are shown in Fig 9. On the left side, the processing time of each method is presented. It is expressed as the necessary time to estimate the position of the robot versus the size of the model (*n* is the number of images in the previously created model). On the right side, the memory requirements of the algorithm versus the number of images in the model is presented.

The method 2 is the computationally most efficient method, and the method 3 is the least efficient one. About the method 1, when it is used along with the *cityblock* or the Euclidean distances, the processing time takes reasonable values, arriving to around *2* seconds when the model is composed of *n* = 2^14^ = 16384 images (the evolution of the time with both distances is the same and the graphical representations overlap). We must take it into account that the objective of this analysis is to study the performance of the methods to solve an absolute localization task. In a real application, when the environment is very large and the model contains a big amount of images, some algorithms can be implemented to carry out the localization process in real time, such as probabilistic localization [14], where the previous position of the robot can be taken into account to reduce the number of images used to solve the image retrieval problem.

## 4. Side detection with respect to a trajectory

In the previous section, a topological localization method with respect to a trajectory-like map has been developed and tested. Initially, the trajectory-like model of the environment is available. This model or map is composed of a set of image descriptors $d_{j}\in R^{360\times N_{x}},j=1,\ldots ,n$ captured from known positions and orientations (*x*_*j*_,*y*_*j*_,*θ*_*j*_). Later, when the robot is at an unknown position (*x*_*t*_,*y*_*t*_) with an unknown orientation *θ*_*t*_ it is possible to identify the nearest neighbour position of the map $\left(x_{t}^{nn},y_{t}^{nn}\right)$ using the methods presented in section 3.1. Also, the relative robot orientation $\theta_{t}-\theta_{t}^{nn}$ can be estimated comparing the test image descriptor *d*_*t*_ with the nearest neighbour image descriptor $d_{t}^{nn}$.

From this information, it is possible to know that the robot is around a specific map position. However, it is not possible to distinguish on which side of the route the robot is located. This is a typical problem when global appearance descriptors are used since they do not contain any metric information, and it can be a serious drawback in case this information has to be used to solve the navigation problem. To solve this problem, the robot should start approaching the route from its initial position and follow it till the target point. If the robot knows (up to an existing uncertainty) the point it is located around but not the side, it is impossible to deduce in which direction it has to start moving to tend to the route.

Taking these facts into account, in this section, we develop an additional method to estimate which side of the route the robot is located on. This method has to be used after the localization and orientation estimation processes exposed in the previous section.

**Fig 10:**
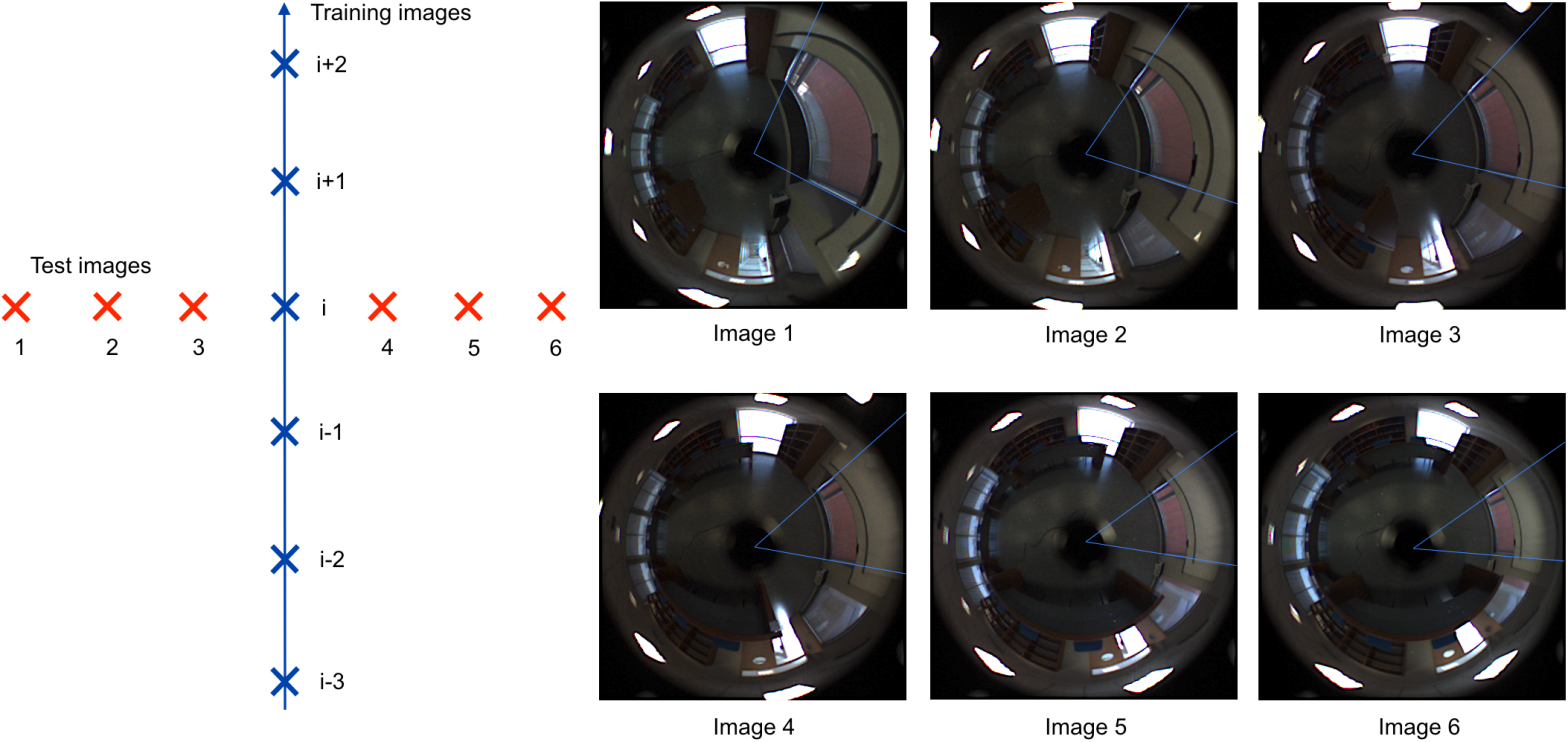
Side detection. Capture points of the test images (red crosses) and the training images or map (blue crosses). The distance between each pair of adjacent images is equal to 40 cm. The omnidirectional images captured from the test positions are also shown.

Let’s suppose a set of test images has been captured around a trajectory-like map. In Fig 10 the test images are shown as red crosses and the linear map as blue crosses on the left side. The right side of Fig 10 shows the omnidirectional scenes captured on each of the test points. If each image is compared with the image situated on its right, it can be clearly appreciated that the objects have moved in such a way that the radii situated in the interval ]0,180[ *deg*. in the first image, tend to decrease their angle, and the radii situated in the interval ]180,360[ *deg*. in the first image, tend to increase their angle. This effect is more noticeable in the case of objects that are situated far from the catadioptric system axis.

Taking this property into account, a method has been developed. This method consists in comparing each column of the RT test image descriptor ***r***_*t*_ (method 3) with all the columns of the nearest neighbour image descriptor $r_{t}^{nn}$. The objective is to know how much each column of the test image RT has to move to match a column of the map image RT. Let’s suppose the image retrieval (localization) problem has been solved with test image *im*_*t*_, according to the previous section. To estimate the side where the robot is, the next algorithm can be run:

1. Calculate the RT descriptor of the test image ***r***_*t*_.
2. Compare ***r***_*t*_ with the RT descriptor of the nearest neighbour $r_{i}=r_{t}^{nn}$ and calculate the relative orientation between them *θ*_*ti*_ = *θ*_*t*_ − *θ*_*i*_.
3. Correct the orientation of ***r***_*t*_ so that its orientation is equal to the orientation of the nearest neighbour. This correction is done as a shift of columns.
4. For each column *θ*_*k*_,*k* = 1,…,360 of ***r***_*t*_.
  4.1. Obtain the most similar column of ***r***_*i*_, using POC as distance measure. With this aim, compare the column *θ*_*k*_ of ***r***_*t*_ with the columns of ***r***_*i*_ which are in the range [*k* − *α*,*k* + *α*]. Let’s suppose the most similar column is *θ*_*j*_, $dist\{\theta_{k},\theta_{j}\}=1-max\{C_{\theta_{k}\theta_{j}}\}$.
  4.2. Retain this correspondence only if *dist*{*θ*_*k*_,*θ*_*j*_} < *c*_*Th*_ and, in this case, calculate the difference $\Delta \theta_{k}^{corresp}=\theta_{j}-\theta_{k}$.

Once this algorithm has finished, the difference $\Delta \theta_{k}^{corresp}$ calculated for each column *k* = 1,…,360 of ***r***_*t*_ will give us an idea of how each radius of the test image *f*_*t*_ has moved to obtain the map image *f*_*j*_. The average difference permits knowing if the test image is on the right or on the left of the map image.

In this algorithm, two important parameters must be tuned. The first parameter is *α*, which defines the range of angles where the column *θ*_*k*_ of ***r***_*t*_ is searched in ***r***_*i*_. This angle is defined around the column *k* in the descriptor ***r***_*i*_ defining the following range: [*k* − *α*,*k* + *α*]. The second parameter is *c*_*Th*_, which is the distance threshold. If the distance between the column *θ*_*k*_ of ***r***_*t*_ and the column *θ*_*j*_ of ***r***_*j*_ is below this threshold, it is considered a good correspondence.

To test the validity of this algorithm, a complete set of experiments has been carried out, using a different database that contains 872 omnidirectional images which have been captured on a dense regular 40 x 40 cm grid of points, covering a whole floor of a building (Quorum V building, 2nd floor) at Miguel Hernandez University, Spain. The whole database is downloadable from [36]. From this database, 20 images have been chosen to compose the training test. Around the capture point of each training image, 3 pairs of images have been selected at distances equal to 40 cm, 80 cm and 120 cm (thus the total number of test images considered is equal to 120). The six capture points are on a line which is perpendicular to the direction the robot had when capturing the training (central) image. Fig 10 shows an example of the six test images capture points around the training image capture point *i*.

**Fig 11:**
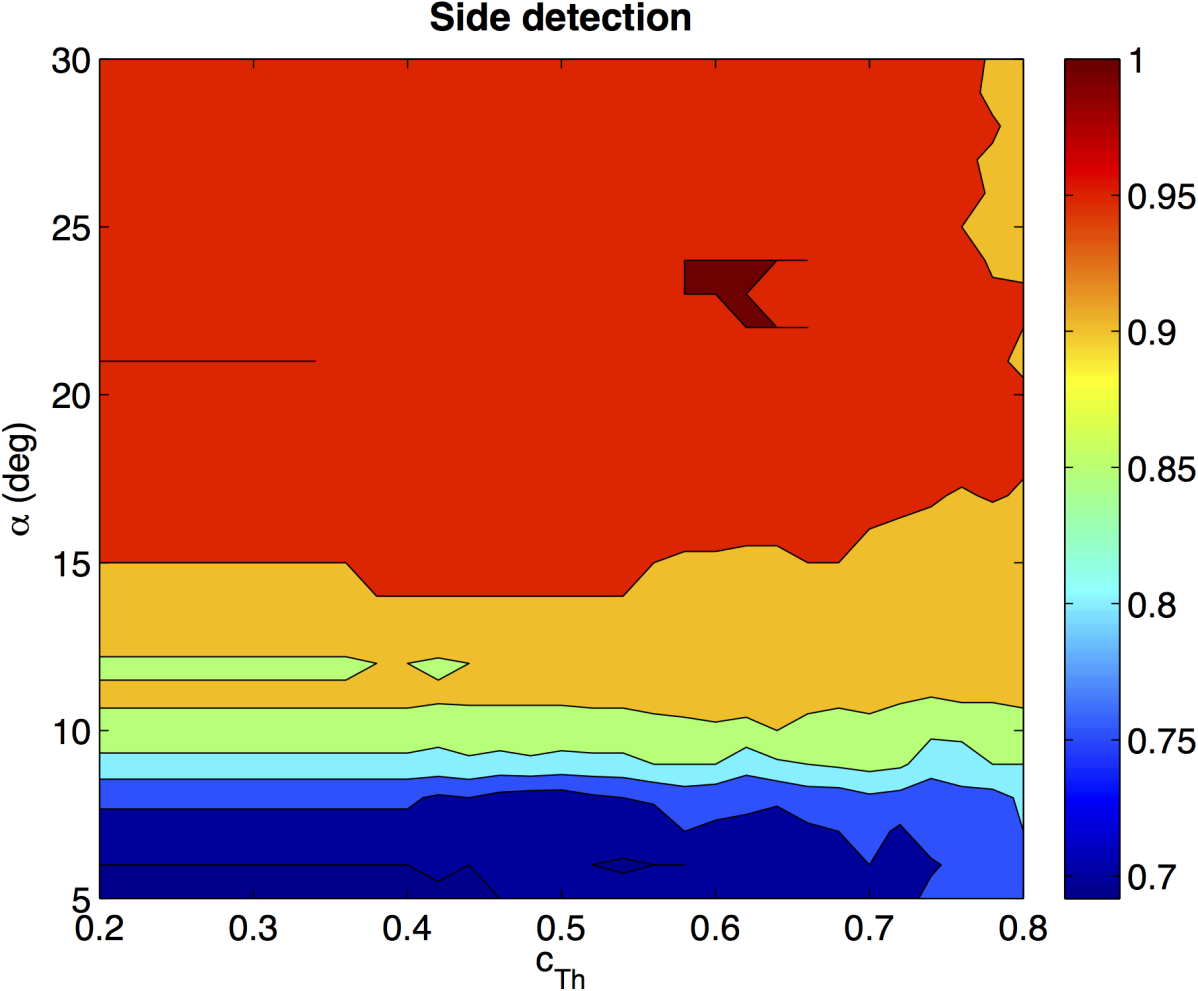
Results of the side detection algorithm. Percentage of test images that have been classified on the correct side of the trajectory versus *α* and *c*_*Th*_.

The algorithm has been run using all the test images to know whether they are situated on the left or on the right of the corresponding training image. The results will be expressed as a percentage of test images that are classified on the correct side (expressed on a per-unit basis). Fig 11 shows the success rate of the algorithm versus *α* and *c*_*Th*_. To carry out this experiment *α* takes the values [5,6,7,…,30] *deg* and *c*_*Th*_ takes the values [0.2,0.22,0.24,…,0.8]. This figure shows that the success rate is quite sensitive to *α*, and it is important to tune correctly this parameter. In general, the algorithm tends to behave quite robustly and it is possible to achieve 100% of correct classification when the parameters take value in the ranges *c*_*Th*_ ∈ [0.58,0.64] and *α* ∈ [22,24] *deg*.

**Fig 12:**
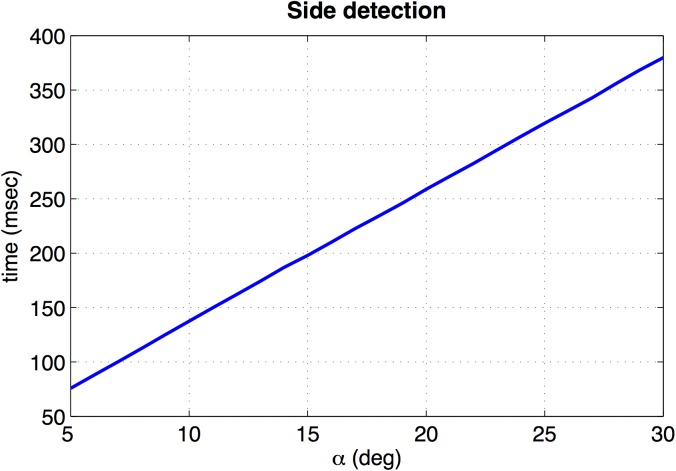
Processing time of the side detection algorithm. The figure shows the necessary time to detect the side versus *α*.

To finish, Fig 12 shows the processing time of the side detection algorithm versus *α* (*c*_*Th*_ has no effect in this processing time). When *α* = 22 *deg*, the processing time is equal to 282 msec.

## 5. Discussion

In real applications, when a mobile robot has to create a model of an unknown environment, it is usual that the robot goes through a trajectory while capturing the visual information to store in the model. This way, it is necessary to work with sets of images captured along a trajectory to create robust maps. These models must be useful to estimate the position of the robot and to allow a visual control so that the robot can navigate using the information stored in the model. This work has focused on these problems and the use of omnidirectional visual imaging and global appearance descriptors to solve them.

First, a description method based on the Radon Transform has been proposed and some methods to obtain and compare descriptors have been presented and tested. A comparative evaluation has been carried out with all these methods to test their robustness to solve the robot localization task. During this process, several distance measures have been considered in the analysis. The performance of the proposed algorithms has been tested under some challenging conditions such as severe noise and/or occlusions in the test images

The Radon Transform has demonstrated to be a robust way of describing omnidirectional images in mapping and localization tasks: the models built with this approach are quite intuitive and the robot is able to estimate its position using only visual information in a straightforward way, based on the pairwise comparison of descriptors. Two sets of images captured by ourselves in different environments and under real working conditions have been used to test the performance of the algorithms. The main contributions of the paper include the study of the localization process using global appearance descriptors and a trajectory-like model, the evaluation of the performance when noise or/and occlusions are present and degrade substantially the appearance of the images, the comparison with two state-of-the-art approaches, and the development of a method to estimate on which side of the map the robot is situated. The experiments have demonstrated that it is possible to find robust solutions to these problems so this work constitutes a good basis for the implementation of a complete visual control framework.

## 6. Conclusion and future research directions

The results presented in this work show the feasibility of the global appearance description methods in mapping and localization tasks and, especially, the robustness of the Radon Transform to create such descriptors, thanks to the integration process it follows to build the descriptor.

The use of the Radon Transform presents some advantages comparing to other global appearance-descriptors (such as the Histogram of Oriented Gradients or *gist*). First, it can be obtained working directly with the omnidirectional image so no further projection is necessary (such as the cylindrical projection to obtain the panoramic image). Also, since it integrates the information of the scenes along some sets of lines to build the descriptor, the new information tends to be robust against noise and occlusions, as the experiments have demonstrated. This means that the descriptor is able to capture the visual information around the robot with robustness. Thanks to it, the robot is able to recognize a localization although the visual information is severely corrupted (with noise and/or partial occlusions). This descriptor also contains useful information to estimate the orientation of the robot with respect to the orientation it had when capturing the images of the map. At last, the mathematical process to obtain it and the new coordinates of the RT descriptor (with angles in the horizontal axis) permits developing a method to estimate on which side of the route the robot is located. This is a relevant feature as it may permit the development of a navigation approach that makes the robot tend to the desired target point. Also, the processing time of the localization process using the Radon Transform (with method 1) is lower compared to Histograms of Oriented Gradients and *gist*.

Once the utility and robustness of such descriptors have been proven, it would also be interesting to develop and implement a probabilistic localization algorithm using global appearance. This way, the information of the previous position of the robot could be used to avoid solving globally the localization problem at each time instant. Furthermore, using the information provided by the localization and side detection algorithms, a visual control framework could be implemented. With this framework, the robot would be able to navigate to the target points in the environment following the routes previously included in the model.
